# A novel *SCN9A* gene variant identified in a Chinese girl with paroxysmal extreme pain disorder (PEPD): a rare case report

**DOI:** 10.1186/s12920-022-01302-z

**Published:** 2022-07-15

**Authors:** Yi Hua, Di Cui, Lin Han, Lu Xu, Shanshan Mao, Cuiwei Yang, Feng Gao, Zhefeng Yuan

**Affiliations:** 1 Department of Neurology, The Children’s Hospital of Zhejiang University School of Medicine, National Clinical Research Center For Child Health, 3333 Binsheng Road, Hangzhou, 310052 Zhejiang Province China; 2 Running Gene Inc., Beijing, 100083 China

**Keywords:** Paroxysmal extreme pain disorder (PEPD), Harlequin color change (HCC), Headache, *SCN9A* gene, Case report

## Abstract

**Background:**

Paroxysmal extreme pain disorder (PEPD) is a rare autosomal dominant hereditary disease, characterized by paroxysmal burning pain in the rectum, eyes or mandible and autonomic nervous symptoms, including skin redness and bradycardia. PEPD is a sodium channel dysfunctional disorder caused by *SCN9A* gene variants. It occurs mainly in Caucasians and only one case has been reported in the Chinese population. Here, we report the second PEPD case in a Chinese indivisual.

**Case presentation:**

A 2 years and 6 months old girl initially presented with non-epileptic tonic seizures at 7 days after birth. Her clinical symptoms in order of presentation were non-epileptic tonic seizures, harlequin color change and pain. Genetic analysis showed the patient carried a heterozygous variant c.4384T>A (p.F1462I) in the *SCN9A* gene, which was speculated to cause PEPD symptoms. After administrating carbamazepine, the symptoms were relieved and the patient's condition improved. However, the patient’s mother, who carries the same *SCN9A* variant as her daughter, only showed bradycardia and sinus arrest but no PEPD-related pain.

**Conclusions:**

This is the second PEPD case reported in the Chinese population. With the discovery of a novel variant in *SCN9A*, we expanded the genotype spectrum of PEPD. This is the first case suggesting that the clinical presentations of *SCN9A*-associated PEPD may show inter familial phenotypic diversity. In the future of clinical diagnosis, patients with triggered non-epileptic tonic seizures or pain and harlequin color change should be considered for PEPD and proper and prompt treatment should be given.

**Supplementary Information:**

The online version contains supplementary material available at 10.1186/s12920-022-01302-z.

## Background

Paroxysmal extreme pain disorder (PEPD or PEXPD, OMIM #167400) is a rare autosomal dominant hereditary disease. In the neonatal and infant periods, the main manifestations of PEPD are non-epileptic tonic attacks, skin flushing, harlequin color change (HCC), bradycardia and other symptoms in autonomic nervous system. In later life, the main manifestation is paroxysmal burning pain in the rectum, eye or mandible. These painful symptoms are often triggered by defecation, perineum stimulation, coldness, eating or mood swings. PEPD was first reported by Hayden and Grossman [1] and named as familial rectal pain by Dugan [2]. In 2005, heterozygous variants in the *SCN9A* gene were reported as a causative factor of PEPD and the name of this disease was finally determined [3]. The *SCN9A*-associated disorder showed 100% penetrance to date [4].

PEPD has been reported mainly in the Caucasian population and only one case has been reported in the Chinese population [5]. Herein, we report a Chinese girl with PEPD symptoms, who also carries a novel *SCN9A* variant. To our knowledge, it is the first *SCN9A*-asscociated PEPD family with phenotypic diversity to be reported.

## Case presentation

**Fig. 1:**
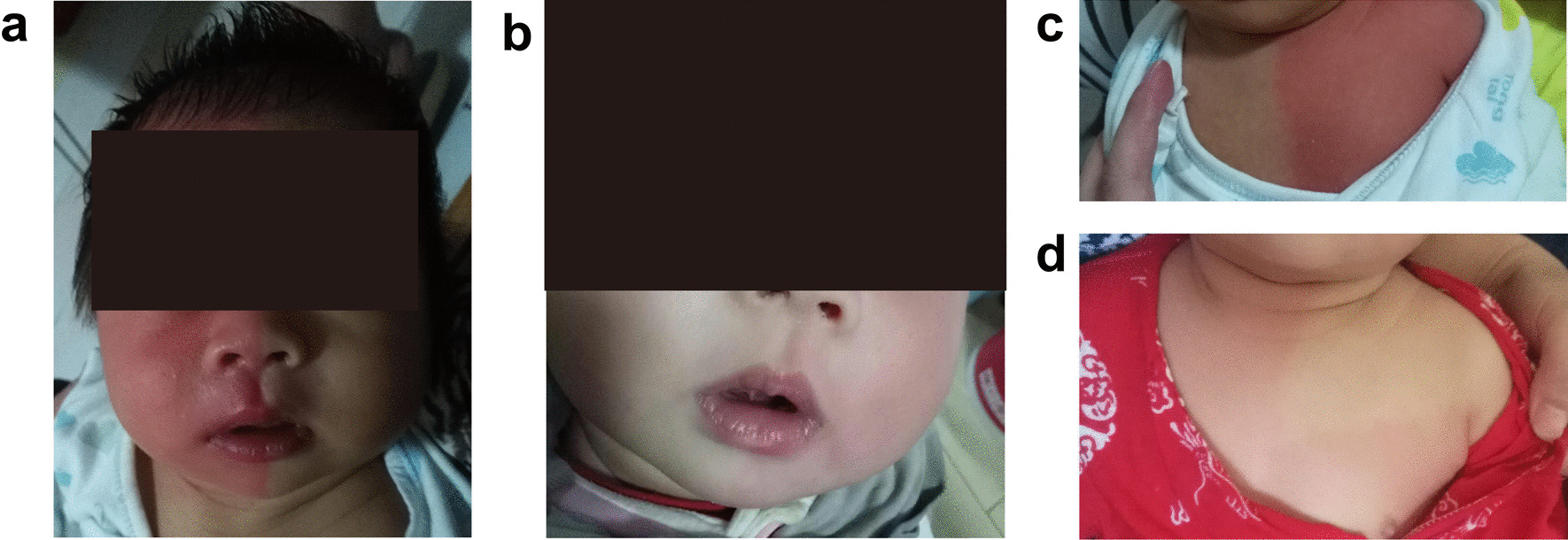
Skin flushing on **a** the right face, **b** left face, **c** left trunk and **d** bilateral trunk.

A 2 years and 6 month old girl presented to our hospital due to recurrent seizures, flushing and sweating of the skin on her unilateral face and trunk and paroxysmal headache. She was a 34-week premature infant with no history of perinatal injury. PEPD symptoms started 7 days after birth. From birth to 3 months of age, she presented with recurrent convulsions with tight-closed or staring eyes, stiff limbs and no response to calls, reddening of the complexion and trunk accompanied with cyanosis of the face and lips. Her non-epileptic tonic seizures were usually triggered by stimulation of the perineum, defecation, or touching the body and usually last for several minutes. From 3 months to 1 year of age, the patient experienced paroxysmal flushing or sweating on the face (Fig. 1a, b) or trunk (Fig. 1c, d), usually occurring several times a day and lasting 20–30 min each time. The flushing was predominantly unilateral, which is known as HCC. Occasionally, the patient showed red flushing at the corners of her eyes when she cried. She was conscious without closed or staring eyes or abnormal changes of the pupil and it usually lasted for approximately 10 min. At two years old, she had a paroxysmal headache, accompanied by local facial or half trunk skin reddening with high frequent attacks. The pain often occurred during eating and could also be triggered spontaneously during sleep. It occurred on the forehead and temporal regions, usually lasting 1–2 h. She also had constipation since infancy. The patient is the first child of her non-consanguineous parents (G1P1). Her mother had a history of syncope, bradycardia, sinus arrest and other arrhythmias so that she was given a pacemaker. She had constipation but denied any history of rectal, eye or mandibular pain since childhood.

Her intellectual development was normal, while her motor development lagged behind that of children of her age. She walked independently at 2 years and 3 months old. When she was admitted to our hospital , no obvious abnormalities were found upon general physical and nervous system examinations. No Horner syndrome or other abnormalities were observed in the pupil. Results of laboratory tests, including blood glucose, electrolyte, hepatic and kidney functions, thyroid function, blood ammonia, blood and urine metabolic screening, were all normal. Video-EEG showed no abnormalities in the interictal and symptomatic phases (autonomic epilepsy excluded). MRI of her head and spinal cord showed no abnormalities. Results of dynamic ECG suggested sinus arrhythmia, temporary sinus bradycardia and temporary sinus arrest.

The administration of carbamazepine (10 mg/kg/day, bid; then, gradually increased to 20 mg/kg/day, bid) reduced the number and severity of attacks obviously. After 3 months of carbamazepine therapy, the girl’s motor ability improved. She could jump forward and backward and run. The patient has been taking the medication for 2 years, resulting in significant pain relief and improved motor ability.

**Fig. 2:**
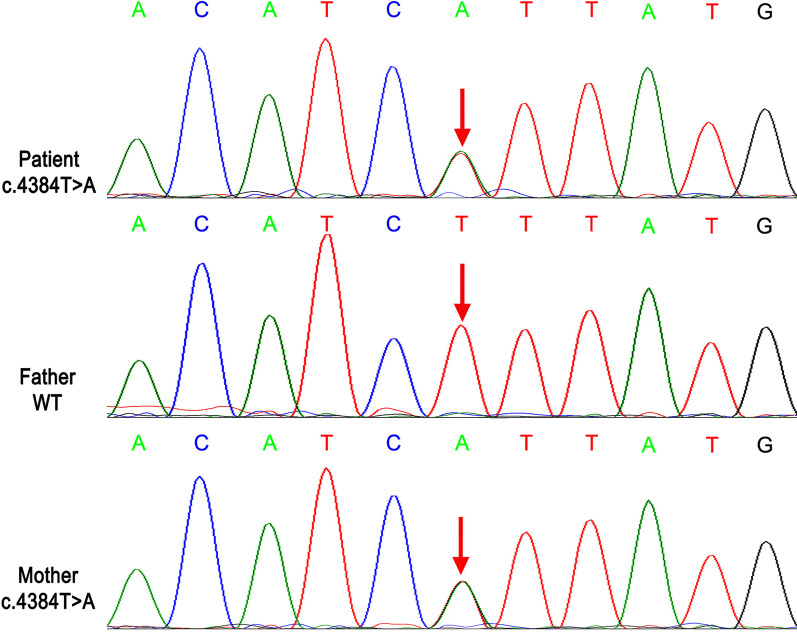
Results of Sanger sequencing on *SCN9A* gene. Both the patient and her mother carry the heterozygous variant c.4384T>A (p.F1462I). Her father does not carry it. Red arrows indicate the site of the variant.

The peripheral blood sample of the patient was collected and sent to Running Gene Inc. (Beijing, China) for whole-exome sequencing. Detailed methods have been described in the previous study [6]. Sanger sequencing was performed on the patient and her parents for confirmation and the co-segregation analysis. Whole-exome sequencing identifed a novel heterozygous variant c.4384T>A (p.F1462I) in the *SCN9A* gene (NM_002977.3), which was inherited from her mother (Fig. 2). The variant is absent from the general population in the databases of 1000 Genomes Project [7], ExAC [8] and gnomAD [9] (PM2). It was predicted to be deleterious by in silico algorithms (REVEL [10], score = 0.964 > cutoff = 0.75) (PP3). Although c.4384T>A (p.F1462I) was novel, c.4384T>G (p.F1462V) has been reported in the Human Gene Variant Database (HGMD) [11] as a disease-causing mutation (DM) at the same site (PM5) [12]. Besides, this variant was in the isoleucine, phenylalanine and methionine (IFM) motif, which forms the inactivation gate and plays a pivotal role in fast inactivation (PM1) [13, 14]. Therefore, the variant identified in our patient was evaluated as likely pathogenic according to the guidelines of the American College of Medical Genetics (ACMG) [15].

## Discussion and conclusions

The major clinical manifestations of neonatal or infantile onset PEPD include non-epileptic tonic attacks (stiffening), skin flushing, HCC and syncope with bradycardia. Patients usually present with apnea, bradycardia and cessation of crying in the neonatal period and apnea after screaming, pale complexion and limb rigidity in the infancy period [16]. Atonic attacks infants can be easily confused with hyperexplexia, reflex anoxic seizures or epileptic seizures, thus video EEG during attacks is required to distinguish PEPD from others. As bradycardia occurs transiently in pediatric patients with PEPD, monitoring of the ECG is also necessary. The patient reported in our study had recurrent non-epileptic tonic seizures in her neonatal and infant periods, with attack frequency decreasing with age. Her symptoms are consistent with that of cases reported previously [16, 17]. The patient’s mother had relatively mild symptoms. She had syncope and unconsciousness with significant bradycardia in her adulthood. Nocturnal asphyxia was still present despite her being fitted with a pacemaker, which may be related to autonomic dysfunction in PEPD.

Skin flushing and HCC are typical clinical symptoms of PEPD. Skin flushing usually occurs in areas of pain. For example, rectal pain is usually accompanied by skin flushing on the pudenda and lower extremities; ocular pain is accompanied by periocular skin flushing. However, there is no absolute correlation between the presence of pain and the site of skin flushing. Some rectal pain may accompanied by facial flushing [17]. Fifteen families have been reported in the Fertleman's study, who had tonic attacks with HCC. The distribution of HCC varies from patient to patient, with most being in the area of pain [17]. In our case, the HCC appeared mainly on the skin of her face and trunk, rather than on the perineum and lower limb skin associated with rectal pain. Occasionally, patients may experience flushing for no apparent cause, a condition that can easily be confused with congenital harlequin syndrome (Table 1). Therefore, for early PEPD, disease differentiation from congenital harlequin syndrome is recommended.

**Table 1:** Differential diagnosis of PEPD and congenital harlequin syndrome.

	PEPD	Harlequin syndrome
Age	Neonate or early infancy	Above one year old (After walking)
Non-epileptic tonic seizures	Yes	No
Pain or panic	Yes	No
Location of the skin flush	Mostly on perineum and lower limbs	Mainly on facial and body cadres
Bradycardia and other autonomic nervous dysfunction	Yes	No
Causes	Defecation and other perineal stimulation, eating, etc.	Temperature, exercise, emotion, etc.

Pain is the primary symptom of PEPD, which is usually localised to the rectum, eye and mandible. However, when the pain is severe, it can spread from the rectum to the abdomen and even throughout the body. Spreading pain is often worse [17]. Pain is most often reported in older children or adults. As infants usually present as frightened, screaming or seeking comfort, their signs are easily overlooked by parents. Therefore, it is necessary to monitor children with the above-mentioned manifestations for pain triggers such as wiping the perineum, defecation, vaccination, eating and medication. With age, the rectal pain will be relieved and the pain will manifest itself mainly in the eyes and jaw [17]. In our case, the patient’s pain symptoms were not observed or recognized by her parents during the neonatal and infant periods. As the patient aged, she developed more pronounced and severe symptoms, such as headaches and autonomic nervous dysfunction, along with skin flushing and bradycardia. In addition, there was a delay in motor development in this patient, which might be associated with pain caused by fear of falling. This symptom has also been described in previous report [18].

*SCN9A* mutations have been reported to be the cause of PEPD [12, 17]. *SCN9A* gene locates on *2q24.3* and encodes the α subunit of the type IX (Nav1.7) voltage-gated sodium channel, which is expressed primarily in the sensory and sympathetic nerves of the dorsal root ganglia of the peripheral nervous system [16, 17]. At first, Fertleman *et al*. identified 8 *SCN9A* missense variants in 11 families and 2 sporadic cases. Functional studies of 3 missense variants showed a reduction in the rapid inactivation of the sodium channel, leading to the persistence of sodium currents [12]. To date, a total of 180 *SCN9A* mutations have been reported in HGMD, of which only 13 DMs are clearly associated with PEPD. Noteworthy, the variant c.4384T>G (p.F1462V) has been identified in a sporadic case with PEPD. Amino acid F1462 is highly conserved (PhastCons, score = 1) and also locates in a highly conserved IFM motif, which was verified to form the inactivation gate and play a pivotal role in fast inactivation by site-directed mutagenesis studies [12]. In our study, the novel missense variant, c.4384T>A (p.F1462I), is located at the same site and presumably affects the normal function of the ion channel by the same mechanism. The patient’s mother, who also carries the same variant, presented with syncope, bradycardia, sinus arrest and other autonomic symptoms that are not typical of PEPD. The mosaicism in the mother was excluded by ultra-deep targeted sequencing on DNA samples obtained from different tissues including blood, urine, hair and oral mucosa, whose ratios between the wild type and variant bases are 0.48, 0.5, 0.49 and 0.49 respectively (Additional file 1: Table S1). The differences in clinical presentation between the patient and her mother demonstrate the phenotypic diversity among family members. Similar intra- or inter-familial phenotypic diversity has been described in *SCN9A*-associated small fiber neuropathy, probably causing by a gain-of-function variant [19]. It is likely that the phenotypic diversity in PEPD we identified here is also caused by gain-of-function of the protein. Gene modifications, the environment or other underlying diseases are all factors that may affect the presentation of PEPD symptoms [20].

The priority aim of PEPD management is to reduce pain symptoms. As a sodium-channel blocker, carbamazepine can reduce the duration of sodium’s current prolongation. This inhibition appears to be dose-dependent [3, 17]. In our study, carbamazepine relieved the patient’s symptoms. Other sodium-channel blockers, topiramate and lamotrigine, have also been reported to be effective in some cases [20]. Other medicines including anticonvulsants, tricyclic antidepressants and serotonin-norepinephrine reuptake inhibitors have also been applied to treat PEPD, although their efficacy is uncertain at this time (20). Reducing pain triggers is another idea to reduce the number and severity of attacks, especially for patients intolerant to medication.

In summary, this study described a Chinese PEPD case and offers us new insights into *SCN9A*-associated PEPD in the Chinese population. To our knowledge, this is the first report of a *SCN9A*-asscociated PEPD family with phenotypic diversity. The novel *SCN9A* variant identified here expanded the mutation spectrum of PEPD. PEPD patients presented with various clinical manifestations at different stages of development. Early clinical identification, genetically assisted diagnosis and carbamazepine treatment are significant for the diagnosis of PEPD and the relief of pain symptoms. In the future of clinical diagnosis, patients with triggered non-epileptic tonic seizures or pain and HCC should be considered for PEPD. Genetic sequencing, especially for *SCN9A* gene, is recommended to clarify the causes of the disease and support the clinical diagnosis.

## Supplementary Information

**Additional file 1. Table S1**. Ultra-deep sequencing of SCN9A from different tissues of the patient’s mother.

## Data Availability

The data analysed in this study is deposited in NCBI Sequence Read Archive (SRA). The accession number is SRR19616526.

## Abbreviations

PEPD: Paroxysmal extreme pain disorder
HCC: Harlequin color change
EEG: Electroencephalogram
MRI: Magnetic resonance imaging
ECG: Dynamic electrocardiogram
ACMG: American College of Medical Genetics
