# Supplementary material for: A novel SCN9A gene variant identified in a Chinese girl with paroxysmal extreme pain disorder (PEPD): a rare case report
Source: BMC Med Genomics. 2022 Jul 15;15:159. doi: 10.1186/s12920-022-01302-z (PMC9287976; doi:10.1186/s12920-022-01302-z)
Supplement: Supplementary file 1 — Additional file 1. Table S1. Ultra-deep sequencing of SCN9A from different tissues of the patient’s mother. [file 12920_2022_1302_MOESM1_ESM.docx]

**Table S1. Ultra-deep sequencing of *SCN9A* from different tissues of the patient's mother**

| Tissue | Target area coverage | Average depth of target area | 20X coverage of target area | WT/Variant  Ratio |
| --- | --- | --- | --- | --- |
| Blood | 100% | 7031.800 | 100% | 1340/1243  0.48 |
| Urine | 100% | 7414.680 | 100% | 16176/16416  0.5 |
| Hair | 100% | 7516.950 | 100% | 16458/15967  0.49 |
| Oral mucosa | 100% | 7456.460 | 100% | 16443/15676  0.49 |
